# Evaluation of Preoperative Hematologic Markers as Prognostic Factors and Establishment of Novel Risk Stratification in Resected pN0 Non-Small-Cell Lung Cancer

**DOI:** 10.1371/journal.pone.0111494

**Published:** 2014-10-31

**Authors:** Tiehong Zhang, Yuanzhu Jiang, Xiao Qu, Hongchang Shen, Qi Liu, Jiajun Du

**Affiliations:** 1 Institute of Oncology, Provincial Hospital Affiliated to Shandong University, Shandong University, Jinan, P. R. China; 2 Department of Thoracic Surgery, Provincial Hospital Affiliated to Shandong University, Shandong University, Jinan, P. R. China; Pulmonary Medicine, China

## Abstract

**Background:**

The aims of this study were to investigate whether the preoperative hematologic markers, the neutrophil-lymphocyte ratio (NLR) or the platelet-lymphocyte ratio (PLR) were prognostic indicators and to develop a novel risk stratification model in pN0 non-small-cell lung cancer (NSCLC).

**Methods:**

We performed a retrospective analysis of 400 consecutive pN0 NSCLC patients. Prognostic values were evaluated by Cox proportional hazard model analyses and patients were stratified according to relative risks for patients’ survival.

**Results:**

During the follow-up, 117 patients had cancer recurrence, and 86 patients died. In univariate analysis, age, gender, smoke status and tumor size as well as WBC, NEU, LYM, PLR and NLR were significantly associated with patients’ prognosis. In multivariate analysis, age, tumor size and NLR were independent predictors for patients’ overall survival (P = 0.024, 0.001, and 0.002 respectively). PLR didn’t associated with patients’ survival in multivariate analysis. Patients were stratified into 3 risk groups and the differences among the groups were significant according to disease free survival and overall survival (P = 0.000 and 0.000 respectively).

**Conclusions:**

We confirmed that NLR other than PLR was an independent prognostic factor. Combination of NLR, age and tumor size could stratify pN0 NSCLC patients into 3 risk groups and enabled us to develop a novel risk stratification model.

## Introduction

Tumor associated inflammation and immunology had been demonstrated to play critical roles in the development and progression of various cancers by facilitating malignant cell transformation, promoting cancer cell proliferation and invasion, and influencing tumor response to comprehensive therapies [1], [2]. Links had been established through the increased risk of pulmonary malignancy that existed in patients with chronic obstructive pulmonary disease (COPD) and pulmonary tuberculosis. Chronic inflammation of the lung indicated both a significant etiologic factor and responsive process to lung cancer [3]. As indicators of systemic inflammatory–immunological process, novel markers including plasma C-reactive protein, the Glasgow Prognostic Score (GPS), the absolute WBC (white blood cell) count or WBC components, and the PLT (platelet) count had been investigated as prognostic and predictive markers in diverse cancers [3], [4]. Pretreatment elevating absolute NEU (neutrophil) count or WBC count and decreasing absolute LYM (lymphocyte) count had been suggested as independent prognostic factors for unfavorable survival in patients with NSCLC [5]. However, the absolute hematologic cell counts could vary under diverse physiological and pathological conditions. Recently, the neutrophil-lymphocyte ratio (NLR), as a new systemic inflammatory–immunological marker for prognosis was superior due to the stability of NLR compared with other hematologic cell parameters. A high NLR had been displayed with increased mortality in various cancer populations, including patients with lung, colorectal, breast, stomach, pancreatic and bladder cancer [6]–[13]. More recently, the platelet-lymphocyte ratio (PLR) was reported to have a similar role in predicting cancer mortality compared with that of NLR. Studies had indicated that the patients who had PLR≥200 had significantly shorter progression-free and overall survivals than those with PLR<200 in patients with epithelial ovarian cancer [14]. PLR was a better prognostic factor for survivals compared to elevated PLT or NLR>2.6. However, it was also displayed that PLR was not superior to NLR in predicting prognosis in breast cancer and colorectal cancer [8],[9]. Furthermore, NLR and PLR were associated with malnutrition, weight loss and hypoalbuminemia as chemotherapy induced toxicity in advanced NSCLC treated with paclitaxel and cisplatin [15].

NLR and PLR are highly repeatable, more stable, inexpensive and widely available. However, there is still no evidence determining whether PLR is associated with survival in pN0 NSCLC patients. The present study aims to determine whether the level of preoperative PLR is associated with the prognosis of operable lung cancer patients, and to verify the role of NLR as a prognostic factor in a larger cohort of completely resected pN0 NSCLC.

## Patients and Methods

### Study population

We retrospectively reviewed our clinical cancer biobank database between January 2006 and December 2009. Inclusion criteria were as follows: patients with data on complete hematologic count including leukocyte subtype, with completely lobectomy or wedge resection, with pathological N0 diagnosis, and with squamous cell carcinoma (SCC) or adenocarcinoma (ADC) histology. Exclusion criteria were as follows: patients with non-curative intent cases, with clinical signs or microbiologically proven preoperative infection, presence of coexisting hematologic disorders, autoimmune disorders, patients on recent steroid therapy and patients with any radio or chemotherapeutic therapies before and after the surgery. Finally we identified 400 patients who had undergone complete resections. All patients had undergone routine preoperative evaluations to exclude contraindications.

### Data acquisition

We investigated the clinical profiles of the patients including patients’ medical notes and laboratory results. The methods and results of the preoperative diagnoses were investigated for each patient. Peripheral venous blood samples were collected between 8 and 10 am within 5 days before surgery and were then delivered to the Department of Clinical Laboratory to have the blood routine tests including the NEU, LYM, and PLT counts. NLR was calculated as neutrophil count divided by lymphocyte count and PLR was defined as the platelet counts to lymphocyte ratio. The histopathological findings were classified according to the World Health Organization, and pathological stages of the disease were described according to the 7th TNM staging system for NSCLC.

### Ethics statement

The study was approved by the Research Ethics Committee of Provincial Hospital Affiliated to Shandong University, Shandong University, China. At the time of surgery, informed written consent for the use of their clinical data was obtained from the investigated patients.

### Follow-up and Statistical analysis

Patients were evaluated every 3 months by CT scans of thorax and abdomen ultrasonography for the first 2 years after surgery and annually thereafter. Survival time was calculated from the day of surgery to the last checkup or death by any cause. Nominal data were analyzed using crosstabs and the Fisher exact test. An independent t-test or Mann–Whitney U test was applied for the continuous variables properly. Nominal data were analyzed using crosstabs and the Fisher exact test. In order to find optimal cut-offs capable of splitting patients into groups with different outcomes, the optimal cut-off points were determined as the threshold value with the joint maximum sensitivity and specificity of the receiver operating characteristic (ROC) curves associated with the patients’ overall survival [16],[17]. Cut-offs allowed transforming continuous into categorical variables. A Cox proportional hazard model was used to identify relevant variables affecting survival. Median values are shown with the 95% confidence interval (CI). Survival curves were constructed using the Kaplan-Meier method and compared using the log-rank test. Statistical analysis was performed with SPSS 18.0 software (SPSS Inc, Chicago, IL). Significance was set at *P* of less than 0.05.

## Results

### Patient characteristics

Altogether 68.0% of the patients were male (272 of 400 individuals). The mean age was 60.8±9.2 years (range from 27 to 84 years). Lobectomies (including sleeve, bronchoplastic lobectomies) were performed in 369 patients, and 31 patients underwent wedge resection. Mediastinal dissection was added in all patients. In the recruited cases, there were 161 (40.3%) SCCs, and 239 (59.7%) ADCs. There were 310 and 90 patients staged as pathologically stage I and II respectively. The median follow-up duration was 46 months (range from 1 to 78 months). During this period, 117 patients had cancer recurrence, and the recurrence sites are mostly locoregional, brain, adrenal gland, and liver. 86 patients died due to cancer causes.

Preoperative hematologic counts of all 400 patients were collected and the absolute numbers of each blood component or their ratio were calculated. Patients’ characteristics, the median values and ranges of WBC, NEU, LYM, PLT counts as well as PLR and NLR of the patients are shown in table 1.

**Table 1:** Clinical characteristic of all 400 lung cancer patients.

Characteristic	Data
No. of patients	400
Age (years)	
Mean±SD(range; median)	60.8±9.6 (27–84; 62)
Gender	
Male/Female	272 (68.0%)/128 (32.0%)
Smoke status	
Never smoker/Smoker	180 (45.0%)/220 (55.0%)
Histology	
SCC/ADC	161 (40.3%)/239 (59.7%)
T stage	
T1/T2/T3	163 (40.8%)/194 (48.5%)/43 (10.8%)
TNM stage	
I/II	310 (77.5%)/90 (22.5%)
Tumor size(0.1 cm)	3.6±2.1
WBC count(×10^9^/L)	6.72±2.03
NEU count(×10^9^/L)	4.22±1.73
LYM count(×10^9^/L)	1.83±0.58
PLT count(×10^9^/L)	232±75
NLR	2.6±1.5
PLR	136.4±57.3
DFS (months)Median/Mean±SD	45.0/42.0±19.0
OS (months)Median/Mean±SD	46.0/45.8±16.1

The cut-off points of the NLR, PLR, WBC, NEU, LYM, and PLT were identified as 3.3, 171, 8.2, 5.66, 1.58, and 190 respectively. Table 2 shows the baseline demographics stratified by NLR and PLR in 400 lung cancer patients. Significant correlation between NLR and age, gender, smoke status, histology, tumor size, and TNM stage (*P* = 0.017, 0.000, 0.000, 0.000, 0.000, and 0.000 respectively) were observed. In terms of PLR, only TNM stage (*P* = 0.014) correlated with PLR. Both NLR and PLR associated with patients’ disease free survival (DFS) (*P* = 0.0020 compared to 0.012) and overall survival (OS) (*P* = 0.001 compared to 0.027) significantly. In addition, the correlation between other hematologic parameters (WBC, NEU, LYM, and PLT) and patients’ characteristics were also calculated and shown in Table S1.

**Table 2:** Distribution of clinical characteristics stratified by pretreatment NLR or PLR.

Characteristic	NLR		*P*	PLR		*P*
	<3.3	≥3.3		<171	≥171	
Age						
<65	195	37	0.017	180	52	0.705
≥65	125	43		133	35	
Gender						
Male	203	69	0	208	64	0.209
Female	117	11		105	23	
Smoke status						
Never smoker	158	22	0	142	38	0.779
Smoker	162	58		171	49	
Histology						
SCC	110	51	0	119	42	0.084
ADC	210	29		194	45	
Tumor size						
<3.5	191	29	0	178	42	0.154
≥3.5	129	51		135	45	
TNM						
I	261	49	0	251	59	0.014
II	59	31		62	28	
DFS (months) Mean±SD	43.8±17.7	35.0±22.1	0.002	43.4±18.2	37.3±21.0	0.012
OS (months) Mean±SD	47.3±14.9	39.7±18.9	0.001	46.7±15.5	42.6±17.7	0.027

### Factors predicting survival: univariate and multivariate analyses

The risking factors for DFS or OS were analyzed using the univariate Cox-regression hazard model. As continuous variables, tumor size, WBC, NEU, and NLR (*P* = 0.000, 0.013, 0.004, and 0.007 respectively) were significant factors for recurrence survival in the univariate analysis (Table 3). Acting as categorical variables, age, gender, smoke status, tumor size as well as WBC, NEU, LYM, PLT, PLR and NLR were predictors of DFS. As for overall survival, younger age, female, never smoker, smaller tumor size, lower WBC, NEU, LYM, PLR and NLR were favorable predictors. Interestingly, PLT didn’t associate with OS as a categorical variable significantly.

**Table 3:** Univariate proportional hazards (Cox) regression analyses according to DFS and OS.

Variables inthe equation	DFS		OS	
	*P*	Hazardratio(95% CI)	*P*	Hazardratio(95.0% CI)
Categorical covariates				
Age(<65 versus ≥65)	0.016	1.559(1.085–2.241)	0.008	1.777(1.162–2.717)
Gender(Male versus Female)	0.017	0.595(0.389–0.910)	0.012	0.514(0.306–0.865)
Smoke status(Never versus Smoker)	0.004	1.756(1.198–2.574)	0.034	1.616(1.037–2.519)
Histology(SCC versus ADC)	0.620	0.911(0.631–1.316)	0.253	0.781(0.511–1.193)
Tumor size(<3.5 versus ≥3.5)	0.000	2.101(1.452–3.042)	0.000	2.511(1.611–3.914)
WBC(<8.2 versus ≥8.2)	0.013	1.684(1.117–2.541)	0.011	1.831(1.149–2.918)
NEU(<5.66 versus ≥5.66)	0.001	2.028(1.339–3.074)	0.000	2.300(1.443–3.665)
LYM(<1.58 versus ≥1.58)	0.041	0.680(0.470–0.984)	0.001	0.494(0.323–0.755)
PLT(<190 versus ≥190)	0.044	1.575(1.012–2.452)	0.140	1.469(0.882–2.446)
NLR(<3.3 versus ≥3.3)	0.000	2.067(1.390–3.072)	0.000	2.570(1.648–4.008)
PLR(<171 versus ≥171)	0.039	1.534(1.022–2.304)	0.003	1.985(1.269–3.104)
Continuous covariates				
Tumor size(0.1 cm)	0.000	1.260(1.171–1.356)	0.000	1.303(1.202–1.414)
WBC(×10^9^/L)	0.013	1.110(1.022–1.205)	0.044	1.104(1.003–1.216)
NEU(×10^9^/L)	0.004	1.144(1.043–1.254)	0.005	1.164(1.047–1.295)
LYM(×10^9^/L)	0.311	0.841(0.602–1.176)	0.017	0.598(0.392–0.911)
PLT(×10^9^/L)	0.403	1.001(0.999–1.003)	0.707	1.001(0.998–1.003)
NLR	0.007	1.130(1.034–1.236)	0.000	1.187(1.081–1.303)
PLR	0.074	1.003(1.000–1.006)	0.016	1.004(1.000–1.007)

In multivariate Cox analysis, NLR (*P* = 0.007), age (*P* = 0.016), and tumor size (*P* = 0.001) were significant factors influencing DFS (Table 4). In terms of OS, the independent predicting factors were NLR (*P* = 0.002), age (*P* = 0.024) and also tumor size (*P* = 0.001).

**Table 4:** Multivariate proportional hazards (Cox) regression analyses according to DFS and OS.

Variables in the equation	DFS		OS	
	*P*	Hazardratio(95% CI)	*P*	Hazardratio(95.0% CI)
NLR(<3.3 versus ≥3.3)	0.007	1.741(1.161–2.611)	0.002	2.075(1.317–3.271)
Age(<65 versus ≥65)	0.016	1.572(1.087–2.273)	0.024	1.636(1.067–2.509)
Tumor size(<3.5 versus ≥3.5)	0.001	1.860(1.275–2.711)	0.001	2.221(1.414–3.488)

**Figure 1:**
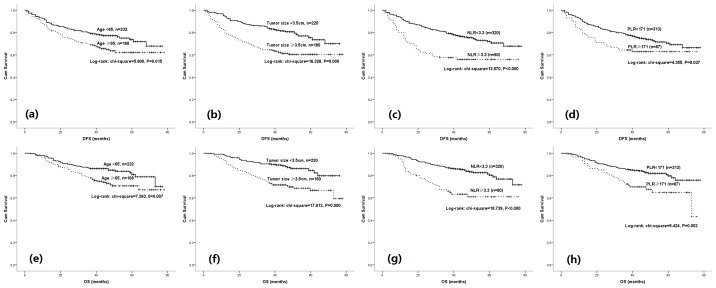
Kaplan-Meier estimates according to categorical age, tumor size, NLR and PLR on DFS (a, b, c, d) and OS (e, f, g, h).

The survival curves according to DFS and OS by age, tumor size, NLR and also PLR were obtained from Kaplan-Meier method and compared using the log-rank test (Figure 1). Clear distinctions in DFS and OS stratified by NLR and PLR were observed (*P* = 0.000, 0.000 and 0.037, 0.002 respectively).

### Risk stratification

**Figure 2:**
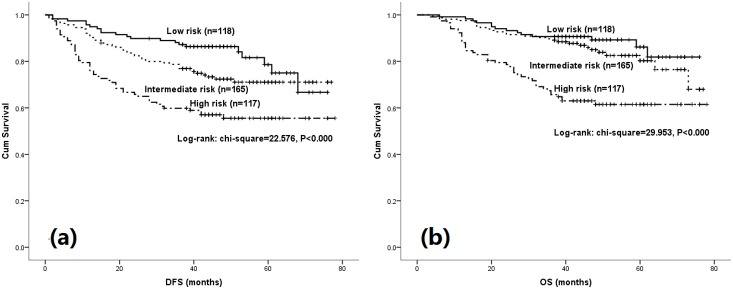
Kaplan-Meier estimates according to low, intermediate and high risk groups on DFS (a) and OS (b).

Using the three statistically significant variables in the multivariate cox regression analysis, the relative risk of survival could be calculated using the formula exp (0.492×age+0.798×tumor size+0.730×NLR). ^14^In this equation, age <65 and age ≥65 equaled to 0 and 1, tumor size <3.5 and tumor size ≥3.5 equaled to 0 and 1, and NLR<3.3 and NLR≥3.3 equaled to 0 and 1, respectively. On the basis of the analysis, the patients were divided into three risk groups: Low risk group which gathered patients with none of the risk factors; Intermediate risk group, which gathered patients with 1 risk factors; High risk group which gathered patients with ≥2 risk factors. Survival curves according to risk groups were shown in Figure 2. From the curves, we could see that clear distinctions between the three groups were observed according to DFS, however no clear distinction was seen between low and intermediate risk group according to patients’ OS. This difference between DFS and OS can be explained by relatively shorter follow-up, and patients who underwent recurrence but not died due to cancer causes accounted a large part in the intermediate risk group. With longer follow-up, the tendency of clear distinction would occur for the patients’ overall survival.

## Discussion

To our best knowledge, the result of our study is the first to show that increasing NLR as a prognostic factor is superior to PLR in resected pN0 NSCLC. Our results indicate that increasing NLR and PLR have impact on survival in patients with pN0 NSCLC in univariate analysis. However, increasing NLR but not PLR does exert an independent prognostic value even after adjustment for age, gender, tumor size, and smoking status in multivariate analysis. PLR is not an independent predictor of survival in pN0 NSCLC. These results are partially consistent with previous observations on the association between hematologic parameters and variety of cancers [5]–[13]. Studies comparing the prognostic value between NLR and PLR found that NLR was superior in breast and gastric cancer [8],[9]. However, studies also found that PLR was superior to NLR in predicting ovarian cancer and pancreatic cancer survival [7],[14]. The results of our study confirmed that NLR was superior to PLR in predicting survival of pN0 NSCLC and validated that an elevated NLR was an independent predictor in a cohort population of treatment naïve pN0 NSCLC.

Increasing NLRs were associated with higher stage and remained an independent predictor of survival in patients with stage I disease in a retrospective review of 178 NSCLC patients undergoing complete resection [18]. Kim et al. [19] showed that increasing preoperative NEU counts and percentages, LYM percentage and NLR were meaningful for predicting survival, but only NLR was an independent predictor in multivariate analysis. Another two studies also indicated that a high NLR may be a convenient biomarker to identify patients with poor prognosis after resection for NSCLC [20],[21]. Limitations of the above 4 studies in operable lung cancer are the small sample sizes. As for advanced lung cancer, Teramukai et al. evaluated a total of 338 chemonaive patients with stage IIIB–IV and found linear associations between pretreatment elevated NEU count and short overall and progression free survival after adjustment for known prognostic factors. The relationship between NLR and overall survival was also found, however, it was to some degree weak and non-linear [12]. Another two studies on advanced lung cancer demonstrated that elevated NLR was an independent predictor of shorter survival in patients, and interestingly, one of the studies also showed that elevated NLR might be a potential biomarker of worse response to first-line platinum-based chemotherapy [10],[11]. Furthermore, NLR and PLR were associated with malnutrition, weight loss and hypoalbuminemia and PLR≥150 was related with the development of toxicity grade III/IV and anemia in patients with advanced NSCLC treated receiving paclitaxel-cisplatin chemotherapy [15].

In our series, increasing NLR was found with elders, male gender, smokers, and larger tumor size in pN0 NSCLC. Elders, male and smokers in China always had chronic or subclinical inflammation with their lung and/or airways, which made increasing NLR higher in these patients. Then increasing NLR would lead to worse survival of these patients. This was somewhat consistent with previous studies. After adjusting for known prognostic factors such as age, gender, smoke status and tumor size, patients with elevated NLR (*P* = 0.002, HR = 2.075) but not PLR presented a decrease in overall survival. In addition, we found that the prognostic value of elevated NLRs was comparable in patients who had SCC or ADC.

Risk modeling by prognostic nomograms or risk class stratification, may provide a powerful tool for individualized outcome prediction and/or stratification of patients. According to the NCCN guidelines, early stage NSCLC was not recommended for chemotherapy and/or radiotherapy after complete surgery. However, some of the patients would suffer from recurrence lately. So if more precise risk stratification were established, then beneficial adjuvant therapy could be given to the high risk group patients to improve the patients’ survival. In the present study, we demonstrated a novel preoperative risk stratification model. The risk-class model proposed herein, which incorporated NLR, age and tumor size in our series, was able to discriminate between patients with low-, intermediate-and high-risk of DFS and OS with striking efficiency, especially for patients’ DFS. Our risk stratification model would contribute to clinical practices, because it would assist identification of a subgroup of patients with unfavorable prognosis that might have the potential benefits of innovative therapies.

Links between tumor associated inflammation and immunology and tumor prognosis had been of great interests [1],[2]. The relationship may be explained via an inflammatory process induced by tumor cells and a tumor promoting process by inflammation. First, the causes of elevated NEU or WBC in cancer patients were likely to be the result of paraneoplastic production of myeloid growth factors such as G-CSF or GM-CSF by cancer cells themselves [12],[22]. Also, elevated PLTs could be caused by the stimulation of megakaryocytes by inflammatory mediators released by tumors or inflammatory cells [23]. Second, preclinical studies indicated that NEUs could stimulate tumor angiogenesis by producing vascular endothelial growth factor, matrix metalloproteinases and elastases [24]. Lymphocytes in tumor microenvironment had significant influence on tumor biology. In NSCLC, elevated tumor infiltrating lymphocytes had been shown to inhibit tumor growth and correlated with a favorable prognosis in cancer [25]. Furthermore, platelets can also produce growth factors i.e. platelet-derived growth factor, vascular endothelial growth factor, and platelet factor4 which could stimulate tumor cells proliferation and adhesion to other cells leading to tumor growth and metastases [26].

## Conclusions

To sum up, the present study demonstrates that NLR is superior to PLR and other hematologic parameters as prognostic factors in pN0 NSCLC patients. NLR, as well as age and tumor size are independent prognostic factors in patients with completely resected pN0 NSCLC. A combination of these easily obtained prognostic factors enabled us to develop a novel risk stratification model, which may aid to discriminate patients with favorable or poor prognosis who might be candidates for multimodality treatment strategies.

## Supporting Information

Table S1 **The relationship between other hematologic markers and clinical characteristics.** Pearson chi-square test was adopted and P values were shown.
(DOC)
