# Supplementary material for: Evaluation of Preoperative Hematologic Markers as Prognostic Factors and Establishment of Novel Risk Stratification in Resected pN0 Non-Small-Cell Lung Cancer
Source: PLoS One. 2014 Oct 31;9(10):e111494. doi: 10.1371/journal.pone.0111494 (PMC4216075; doi:10.1371/journal.pone.0111494)
Supplement: Table S1 — The relationship between other hematologic markers and clinical characteristics. Pearson chi-square test was adopted and P values were shown. (DOC) [file pone.0111494.s001.doc]

Table S1 The relationship between other hematologic markers and clinical characteristics. Pearson chi-square test was adopted and P values were shown.

| Characteristic | WBC | NEU | LYM | PLT |
| --- | --- | --- | --- | --- |
| Age | 0.244 | 0.837 | 0.425 | 0.003 |
| Gender | 0.000 | 0.000 | 0.118 | 0.148 |
| Smoke status | 0.000 | 0.000 | 0.565 | 0.535 |
| Histology | 0.000 | 0.000 | 0.676 | 0.292 |
| Tumor size | 0.000 | 0.000 | 0.154 | 0.024 |
| TNM stage | 0.000 | 0.000 | 0.639 | 0.033 |
